# Parenting Styles, Coparenting, and Early Child Adjustment in Separated Families with Child Physical Custody Processes Ongoing in Family Court

**DOI:** 10.3390/children8080629

**Published:** 2021-07-24

**Authors:** Mónica Pires, Mariana Martins

**Affiliations:** 1 Psychology Research Center (CIP-UAL), Universidade Autónoma de Lisboa, 1169-023 Lisboa, Portugal; 2 William James Center for Research (WJCR), ISPA Instituto Universitário, 1149-041 Lisboa, Portugal; mariana.g.c.martins@hotmail.com

**Keywords:** parenting styles, coparenting, early child adjustment, separation, divorce, sole/joint physical custody, family court

## Abstract

Coparenting conflict and triangulation after separation or divorce are associated with poorer child adjustment when parenting gatekeeping and conflict occur. Fewer studies reported psychosocial adjustment of children under three. We explored the effects of authoritarian and permissive parenting styles and negative coparenting on child adjustment in a purpose sample of 207 Portuguese newly separated/divorced parents (50.2% mothers/49.8% fathers) with sole or joint (49.8%/50.2%) physical custody processes ongoing in court. Parents filled out the Parenting Styles Questionnaire—Parents’ report, the Coparenting Questionnaire, and the Strengths and Difficulties Questionnaire. Parenting and coparenting moderations path analysis to predict child adjustment were tested for two groups (2/3-year-old child/>3-year-old-child) and showed a good fit, followed by multigroup path analysis with similarities. Findings suggest harsh parenting and interparental conflict and triangulation as predictors for poor early child adjustment. The ongoing custody process could contribute to increased interparental conflict. The families’ unique functioning, parenting, and conflict should be considered in young children custody decisions made in a particularly stressful period when the parental responsibilities’ process is still ongoing and conflict may increase to serve the best interest of the child and promote healthy development. Future directions and practical implications are discussed.

## 1. Introduction

Divorce or parents’ separation requires a multitude of family and individual adjustments to the new family configuration, structure, and roles on psychologic, social, and economic levels, often involving individual and family adjustments and changes at home, school, in relationships, and even community. Previous studies focused on the aftermath of separation or divorce, but few examined new divorcees. They addressed individual adaptation, parenting, [1], and marital negotiation strategies as predictors of parental alliance [2], parenting time, interparental conflict, and quality of parenting [3]. To our knowledge, no study focused on the transition period when the child physical custody process is still ongoing in court. This demanding, stressful phase is prone to lead to escalating interparental conflict, triangulation, and gatekeeping in parents with children at different developmental stages, particularly in earlier development. This study aimed to test the association between parenting styles and negative coparenting covariates on a child’s adjustment in early development when the child physical custody process is still ongoing in the Family and Minors court.

### 1.1. Parenting and Coparenting after Divorce or Separation

Prior interparental relationships, social support, parents’ psychological traits and health, as well as child characteristics contribute to good family adjustment after divorce [4]. Throughout this period, parents may feel grief, more vulnerable, overloaded with chores and responsibilities and may experience more stress, thus becoming less responsive to their children’s needs [5]. This situation may affect cooperating capacity to communicate and collaborate with the ex-spouse in child rearing responsibilities and parenting styles, which may become less responsive or inconsistent. In most cultures, authoritarian, permissive, or inconsistent parenting styles are associated with poorer psychological adjustment in children, showing more externalized and internalized problems [6,7,8,9]. For instance, nonresident fathers, a common scenario, were found to be less involved and more permissive towards their children, while authoritativeness was associated with children’s well-being in different custodial arrangements [10].

Post-divorced or separation parenting and coparenting play significant roles in parents’ wellbeing and children’s psychological adjustment [11,12], which can have a long-lasting effect [13]. Negative coparenting, such as restrictive gatekeeping [14], conflict, and triangulation has a direct effect on children’s mental health due to their exposure to and involvement in conflict and an indirect effect by affecting parenting and child rearing practices [15]. Conflict increases the risks of negative outcomes for children [16,17] and lower quality of life [18]. If interparental cooperation with frequent communication regarding child rearing has positive outcomes in child adjustment, conflict, triangulation, and more restrictive gatekeeping have a negative impact. In a Norwegian longitudinal cohort study, authors found that fathers with higher education qualifications were more conflictual towards the ex-partner but still present in children’s lives [19]. High conflict parents tend to adopt inconsistent parenting styles, and their children are more likely to present more internalizing and externalizing problems [15]. Considering family complexity, the effect of post-divorced or separation parenting may not be enough to solely predict child adjustment. Some studies found no differences in child adjustment as a result of different coparenting types, emphasizing the role of parenting styles and the quality of the parent–child relationship [20]. In an Australian longitudinal cohort study, one in three households were possibly affected by interparental conflict beyond a six-year period from early development to middle childhood with more frequent verbal conflict than physical [21].

Negative destructive coparenting is associated with lower psychological adjustment in children and adolescents [22,23]. Changes in the dimensions of conflict (frequency, intensity, overt/covert conflict, and conflict resolution) correspond to fluctuations in the child’s emotional reactions and security both in the short and the long term [24,25]. Frequent situations of conflict lead to intense emotional responses in children, namely feelings of sadness, irritation, concern, shame, and abandonment [26]. Lower parental support and communication coparenting are also associated with internalized problems [27]. Previous studies found that parenting higher conflict predicts increased internalized and externalized problems in children over four [28]. Interparental conflict was also associated with low coping efficacy mediated by mother–child relationships [29], inconsistent parenting, and low life satisfaction [15]. Therefore, interparental conflict may be understood in an integrated model whereas family processes, parenting, and individual, parents’, and children’s factors underlie the pathways to child adjustment, regulatory processes, psychological problems, and mental health in separation or divorce and post-divorce phases [30] and in different household arrangements [31].

### 1.2. Child Physical Custody

Increasing divorce and separation rates along with joint child physical custody arrangements justify the development of studies focusing on the impact of coparenting and parenting styles in child adjustment, particularly if the child is under three years old.

In 2019, the divorce ratio in Portugal was 2.0 per 1000 inhabitants, which was within the European Union average. However, these figures do not include marital union dissolutions. In 2019, there were 67,776 paternal single-parent families and 391,568 maternal single-parent families, highlighting the higher frequency of children living exclusively with their mothers. There are no publicly available statistics of sole or joint physical custody [32].

In Portugal, except in cases of rape, violence, abuse, neglect, abandonment, parental absence, or strong controversy between parents, both parents have the right and the duty to exercise parental responsibilities on upbringing, development, and well-being of their children. The law establishes as a rule the joint exercise of parental responsibilities regarding issues of particular importance in the life of the child regardless of the previous type of marital relationship or the type of residence of the child (sole or joint physical custody) (e.g., decisions regarding name of the child, place of residence, school, religion, surgical interventions, traveling abroad, risky sports, work activity, voluntary interruption of pregnancy until 16 years of age, celebration of marriage, and driving license for motorcycles at 16 years of age). In every other decision, parents have the autonomy to decide on the child’s daily life matters.

Joint physical custody is more frequent in children whose parents have higher educational and socioeconomic levels [4]. Even in joint physical custody, asymmetric care arrangements and overnights are more frequent. The quality of interparental relationship and the parents’ working hours was found to positively predict symmetric joint physical custody plans [33]. Some studies found equal levels of well-being in children from both types of child physical custody, similar to those from non-separated families [17,31,34] or sole vs. joint physical custody, emphasizing the role of low parental psychopathology and high parenting and coparenting [35]. Others observed lower adjustment scores in children living with both parents in joint physical custody compared to children living with one parent [36,37,38] and fewer psychosomatic problems mediated by the quality of the mother–child relationship [36]. In a longitudinal cohort study, children in joint physical custody showed slightly different scores compared to sole physical custody [4]. In a large study in Germany, children in joint physical custody presented higher wellbeing when compared to the sole custody group. However, differences in child adjustment disappeared when controlling for parent, child, separation, and quality of family relationship [39]. When high interparental conflict occurs, children from both custody arrangements denote similar levels of mental health problems [37].

### 1.3. Child Sole/Joint Physical Custody, Interparental Conflict and Young Children’s Outcomes

Children’s age and abilities to process separation, divorce, or conflict are relevant for understanding child outcomes. The negative impact of harsh or less responsive parental styles in children differs regarding parent and child gender [40]. Preschoolers’ negative internal representations of parent–child relationships were found to moderate the impact of parental separation and explain conduct problems at the age of six [41]. Preschool-aged children may feel fear, threat, and self-blame as a self-regulatory mechanism, seeking emotional security in parental relationships [42]. Young children (younger than three) do not have the cognitive or the emotional ability to process interparental conflict as preschool children and being unaware could protect them from negative outcomes [30]. They also have limited coping strategies, since toddlers look at adults (such as an attachment figure) for security and as a model.

Sensitive to parents’ distress, when experiencing marital destructive conflict, infants [42,43] and toddlers [44] exhibit more negative emotional reactions or withdraw, which diminish when there is conflict resolution. Destructive conflict is associated with paternal detachment for both parents, lower paternal sensitive parenting, and lower infant emotional security, emphasizing differences in conflict impact according to parental role [45]. In the emotional security hypothesis [46], conflict exposure and lower levels of emotional security in infancy predict child maladjustment in preschool years (lower levels of prosocial behavior and higher conduct problems) and linger beyond conflict exposure [42].

Some studies found frequent overnights to be associated with insecurity of attachment, low well-being, and behavior problems in children under five years old [47,48]. Interparental conflict may affect post-separation visits and nonresident parent involvement in early stages of development (infants and toddlers) [49]. Frequent overnights of children under three were associated with more problems when compared to older children (4- to 6-year-olds, 1.5 year later). Besides the number of overnights, their inconsistency was associated with child negative outcomes, with frequent overnights and multiple caregivers benefiting girls more than boys [50]. Child physical custody and overnight decisions should consider several factors, such as child safety and protection, child health and characteristics, parents’ mental health, previous contact with the parent, attachment and interaction with each parent, along with other circumstances such as house commute [51]. In abusive, noninvolved, neglectful parenting, joint physical custody, or frequent overnights do not protect the child but exposes her to a damaging household environment and risk. Additionally, high conflict parents struggle to communicate effectively and coordinate childrearing relevant decisions. In such cases, joint physical custody may endure conflict and child exposure and probable negative outcomes.

This position is not consensual and is subject to criticism [52,53] and debate [54,55]. In other study with retrospective reports, frequent overnights with a nonresidential parent (usually the father) when the child was two years old, or an acceptable number of overnights contributed to a closer parent–child relationship later in adolescence [56]. Authors highlight the importance of overnight for embracing the fathers’ caregiving role, the closeness of parent–child relationships, and child attachment to both parents, which day visits cannot compensate. Still, mutual agreement consent on overnights and child rearing conditions in both homes may not be recommended immediately after separation or divorce or when one parent was already uninvolved. Following separation or divorce, restrictive gatekeeping, conflict, and triangulation may increase, placing infants, toddlers, and preschoolers in vulnerable conditions or even risk. Restrictive gatekeeping, when the child is placed in the middle of parents’ conflict and triangulation, may easily occur with a young child if the other parent is considered unfit or uninvolved based on pre divorce experience [14]. Due to lack of confidence in ex-partner rearing abilities’ or as a conflict strategy, it may have negative outcomes for the child.

Although the association between negative coparenting, conflict, and lower child adjustment to both internalized and externalized problems is well established [8,27,57,58], very few studies examined these variables regarding young children [42,45,49], particularly during child physical custody process in court. Child outcomes can be understood within the mutual interaction between family systems, parenting, and coparenting in a spillover effect [30]. The moderating effects of parenting styles, coparenting, interparental conflict, and type of custody arrangements in an early stage of development need be addressed.

### 1.4. The Present Study

This study intended to contribute to the research on the impact of coparenting after separation or divorce on child adjustment in a sample of newly separated or divorced Portuguese parents. We intended to explore the effects of parenting styles and negative coparenting on psychosocial adjustment of young children of separated parents with an ongoing litigation or friendly child physical custody process in family court. Post-separation interparental conflict is relevant for the child’s mental health in both custody arrangements [33,37]. First, we intended to describe child adjustment at a young age in the middle of a custody process in court, which is a sensitive post separation phase bound to increase interparental conflict. We hoped to provide useful information concerning this unique sample group—parents of toddlers currently with an ongoing child custody process court—for the body of literature on this matter. Based on previous studies that demonstrated a joint effect of parental and interparental variables on child adjustment [30], we first tested the correlational hypotheses necessary to integrate the covariates in the moderation model, namely, between authoritarian and permissive parenting styles, conflict, and triangulation negative coparenting and negative child adjustment indicators. Finally, we tested their predictive value for child adjustment and compared the model invariance in two groups of parents, (1) 2- and 3-year-old children and (2) children older than three, thus contributing to research on interparental conflict in early development [43,44,45]. The proposed moderation research model in which authoritarian and permissive parenting styles and coparenting through triangulation and conflict predicts child adjustment, namely, through hyperactivity, conduct, relational problems, and emotional symptoms, is well established in the literature focused on post-divorced or separated families with school-aged children or adolescents. Testing it with a sample of separated or divorced parents in a particularly distressed phase for all family members when the child physical custody process is still ongoing in Family and Minors court was the outreach of the current study. Additionally, the evaluation of the predictive effect of the covariates, i.e., parenting and coparenting variables in child adjustment in two groups (parents with a 2- or a 3-year-old child and older than three), intended to test the fit of the theoretical model of the data, filling the gap in research in this particular developmental phase.

## 2. Materials and Methods

### 2.1. Participants

A purpose sample of 207 Portuguese newly separated or divorced parents, 50.2% mothers and 49.8% fathers from 20 to 60 years old (*M* = 37.31; *SD* = 8.27), mostly employed (93.7%) with high school (49.3%), university degree (28.5%), or incomplete education (22.2%) were recruited in a Family and Minors court. Parents of 1 to 2 children answered questionnaires regarding the youngest with the ongoing child physical custody process in court. Children were between 24 months and 16 years of age (*M* = 7.04; *SD* = 4.53), 56.8% boys and 43.2%, girls. Most parents were divorced by mutual consent (76.8%), and a few were in a litigious situation (23.2%). All had an ongoing child physical custody process in the Family and Minors Court from 12 up to 40 months, from which 49.8% were sole and 50.2% were joint physical custody cases. Despite the child custody group equivalence, families practiced an asymmetric care arrangement whereby the child spent, on average, 20.88 (*SD* = 6.40) days/month with the mother and less time with the father 9.04 (*SD* = 6.42). The group of parents of children 2 or 3 years old (57.1% boys and 42.9 girls) consisted of 93 parents (49 mothers; 44 fathers) who were mostly employed (90.3%) and had a high school qualification (50.5%) or college degree (43.1%). Child custody processes included 45.2% joint and 44.8% sole physical custody, of which 87.1% were amicable by mutual consent and 12.9% were in litigation. Children stayed, on average, 9.71 days with their father and 20.29 days with their mother.

### 2.2. Measures

Parent and child information, separation or divorce, and child custody information were gathered in the sociodemographic sheet. Items included separation/divorce time, mutual agreement or litigious process in court, sole or joint physical custody, days per month spent with each parent, and communication forms.

Parenting styles were measured with the Portuguese version of the Parental Authority Questionnaire [59] from Buri (1991), adapted for the parents’ self-report (PAQ-P) [40]. Based on Baumrind’s model [60], it comprises 30 items answered in a 5-point Likert scale, resulting in three composite scores (10 items per PS): authoritarian (e.g., “Whenever I tell my children to do something, I expect them to do it immediately without any questions”), authoritative (e.g., “I guide my children activities and decisions through reasoning and discipline”), or permissive (e.g., “Most of the time I do what my children want when establishing family decisions”) parenting styles. The authoritarian and the permissive scales used in this study had adequate Cronbach’s alpha, assuring adequacy to this particular sample (α = 0.91; α = 0.60).

Coparenting styles were measured with the Portuguese version of the Coparenting Questionnaire (CQ) [58,61]. This 14-item tridimensional scale provides, through a 5-point Likert scale, perceptions about the other parent’s responsibilities. Scores of the 3 main strategies were obtained: cooperation (5 items, e.g., “(…) tells me a lot about our son”) when support and respect for each other exists; triangulation (4 items, e.g., “(…) says cruel things about me, or things that hurt me, in front of our son/daughter”) when an alliance with the child is identified, undermining the other parent’s image or authority; and conflict (with 5 items, e.g., “(…) and I have different rules regarding food, daily routines, bedtime or homework for our child”) when interparental conflict occurs in the children’s upbring. For this study, we only used negative coparenting scales, triangulation, and conflict, which presented good Cronbach’s values, attesting the scale adequacy to our sample (α = 0.98; α = 0.95), with higher scores on conflict and triangulation indicating the possibility of higher interparental destructive conflict.

Psychosocial child adjustment was measured using the Portuguese version of the 25 items Strengths and Difficulties Questionnaire (SDQ) parent-report [62,63]. Sub-scales of hyperactivity (e.g., constantly fidgeting or squirming), conduct problems (e.g., often fights with other children or bullies them), emotional symptoms (e.g., often unhappy, downhearted, or tearful), and interpersonal problems (e.g., rather solitary, tends to play alone) can be operationalized for externalized (adding hyperactivity and conduct problems scores) and internalized problems (adding emotional and interpersonal problems score). For this study, only negative scales were included with Cronbach for emotional, relationship, conduct problem, and hyperactivity values proven suitable (α = 0.88; α = 0.67, α = 0.64, α = 0.63).

### 2.3. Procedures and Analytic Strategy

After project evaluation, research ethics committee approval, and tutelage approval by the Family and Minors Court, data were collected during the parents’ visit to court regarding the child physical custody process and the parental responsibilities’ regulation. Complying with the Helsinki Declaration, data protection rules, and assuring all ethical standards, parents filled out a consent form and willingly answered paper-and-pencil questionnaires regarding their common child as the only child or, in cases with more children, the eldest child. We recruited all participants in court in a specific time frame. We included in our sample all Portuguese parents that attended Family and Minors Court due to an ongoing child custody process that accepted participation and consent. The data collection was completely independent of the ongoing legal proceedings. No financial compensation was given.

For the analytic strategy, descriptive and internal consistency analysis of all measures were conducted, followed by a correlation matrix for two groups (parents with two- or three-year-old children and parents with children over three). To test the conceptual model guided by previous research, including negative parenting styles and coparenting on child adjustment, we performed path analysis, followed by multi-group path analysis within the structural equation model (SEM) framework to verify pathways and model invariance between the two groups of parents. The data were analyzed using SPSS v.25 (IBM, Inc., Armonk, NY, USA), and path model and multi-group analysis were estimated using AMOS complement.

We used maximum likelihood (ML) and revised model specifications according to modification indexes for estimating the model. Finally, we used bootstrap for confirmatory multi-group analysis. Multigroup model analysis was conducted in steps, starting with a baseline model in which parameters were estimated for total sample/both groups with no equal constraints imposed and followed by restrictive models to compare model invariance and testing equivalence across groups.

The evaluation of goodness-of-fit of the path model to the data included multiple indices: Chi-square and degrees of freedom (χ^2^/*df*) (under 5 to consider a good fit); Tucker–Lewis index (TLI), normed fit index (NFI), comparative fit index (CFI) estimates equal/over 0.95; standardized root mean square residual (SRMR) and root mean square error of approximation (RMSEA) below 0.08 for good fit [64,65].

## 3. Results

### 3.1. Descriptives: Parenting Styles, Coparenting, and Child Adjustment

Table 1 provides descriptive statistics and bivariate correlations of parental and child variables reported by parents. Separated parents with children over three encompassed a larger age range and perceived themselves as more authoritarian than permissive in interparental conflict and triangulation. Parents with younger children showed less of these perceptions. For testing hypothesis regarding basic associations between variables under study, we conducted correlations within parenting and child adjustment variables for the two groups of separated or divorced parents with 2- or 3-year-old children and children older than 3 (Table 1). For both groups, we confirmed correlations between parenting styles and negative coparenting, although higher permissiveness was associated with lower triangulating and higher interparental conflict. Higher authoritarian was positively associated with both negative coparenting styles. Additionally, more authoritarian, triangulation, and conflict were significantly strongly correlated with higher perceptions of relationship, emotional, and conduct behavioral problems and hyperactivity in both groups of parents, confirming all the correlation hypotheses. In contrast, correlations between permissiveness, triangulation, and child adjustment indicators were significantly negative, suggesting the protective effect of this parenting style for both age groups.

### 3.2. Path Analisys

Finally, we tested the predictive value of parenting and coparenting styles for child adjustment and compared the model invariance in two groups of parents—those with two- or three-year-old-children and those with children older than three—contributing to research on interparental conflict in early development [43,44,45]. The proposed research model in which authoritarian and permissive parenting styles and coparenting through triangulation and conflict affect child adjustment (namely hyperactivity, conduct, relational problems, and emotional symptoms) is well established in existing literature focused on post-divorced families with school-aged children or adolescents.

In our hypothesis, we included in the path model authoritarian and permissive parenting styles, conflict, and triangulation coparenting as covariate predictors of lower child adjustment for both groups with younger and older children. After determining the sample size for effect (minimum of *n* = 87 for *p* < 0.05), we ran a baseline path analysis model with no constraints for each group separately and gradually imposed restrictions to test invariability across groups, implying a non-significant chi-square difference. Testing the path model in two groups of parents (1) parents with 2- or 3-year-olds and (2) children older than 3, we examined the model fit for both: Group 1 *X*^2^_(3)_ = 2.832, *p* = 0.04; CFI = 0.99; NFI = 0.98; TLI = 0.89; RMSEA = 0.14 (90% CI [0.03, 0.25]); SRMR = 0.03; and Group 2, *X*^2^_(3)_ = 10.462, *p* < 0.001; CFI = 0.98; NFI = 0.98; TLI = 0.81; RMSEA = 0.21 (90% CI [0.15, 0.28]); SRMR = 0.02. Considering fit indexes for both samples, the chi-square significant value implying differences between the sample covariance matrix and the reproduced covariance matrix was comprehensible in larger samples. Higher RMSEA values found in both groups were not desirable. However, the other model-fit indices criteria indicated a good fit to the data.

### 3.3. Multigroup Analysis of Model Invariance

**Figure 1:**
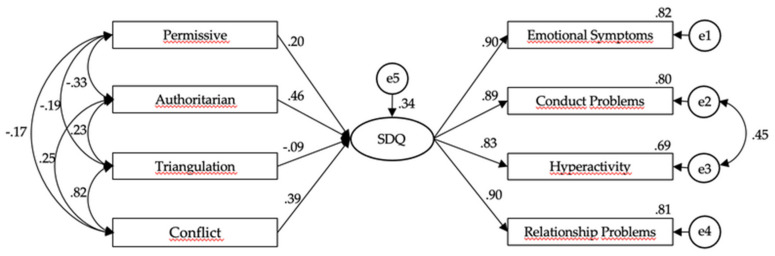
Multigroup analysis of the moderation model for the overall sample. Summary of estimates from authoritarian and permissiveness, triangulation, and conflict to child adjustment (SDQ as a latent variable). Note: Model χ^2^/df = 5.448 (*p* < .01); comparative fit index (CFI) = 0.97; Tucker–Lewis index (TLI) = 0.94; standardized root mean square residual (SRMR) = 0.03; root mean square error of approximation (RMSEA) = 0.10 (90% CI [0.08, 0.13]). For CFI and TLI, a value larger than 0.9, SRMR values below 0.08, indicated good model fit.

Testing our final hypothesis in the multigroup comparison and expecting multigroup model invariance, a final constraint measurement and structural weights model was conducted with a good fit for the data (Figure 1): *X*^2^_/df_ = 5.448, (*p* < .01); CFI = 0.97; NFI = 0.97; TLI = 0.94; RMSEA = 0.10 (90% CI [0.08, 0.13]) with SRMR = 0.03 and with the root mean square error of approximation with a borderline value. Table 2 summarizes the results of the invariance analysis of several models tested. Similarly, for the models for groups 1 and 2, all presented significant chi-square. Nonetheless, the other model-fit indices were acceptable, especially for measurement (CFI = 0.96; SRMR = 0.04; RMSEA = 0.09) and structural weights (CFI = 0.95; SRMR = 0.05; RMSEA = 0.09). Table 3 summarizes the results of the invariance of models under examination, testing for equality of variance/covariance matrices. A non-significant chi-square value suggested invariance in measures and structure weights between groups but not for covariates (Table 3). To the exception of significant chi-square/df models, other criteria suggested good fit for measurement and structural weights, and parsimonious values were lower: model 1 tested baseline unconstraint (good fit for CFI = 0.96 and SRMR = 0.47 but high RMSEA and lower PCFI), model 2 tested measurement weights (good fit for CFI = 0.96 and SRMR = 0.48, better PCFI = 0.49 and lower RMSEA = 0.09), model 3 tested structural weights (good fit for CFI = 0.95 with higher PCFI = 0.56, RMSEA = 0.09 still borderline, and acceptable SRMR = 0.05), and model 4 tested the equality of variance/covariance matrices with only two indices acceptable (CFI = 0.93 and PCFI = 0.71). It did not satisfy the requisites for a good model and had weak fit-criteria values for structural and measurements residuals. A significant chi-square value suggested some variance between groups. As such, parenting and coparenting younger children within an ongoing child physical custody process in court may have different outcomes or poorer adjustment. While comparing models with more constraints, the model fit decreased (Table 3), indicating the covariance activity constraints. Considering previous permissiveness correlations values, one might ponder that permissiveness, authoritarian., triangulation, and conflict may interact differently in different age groups. Thus, we partially confirmed our hypothesis, as the theoretical model could fit both sample groups but with differences between them. These chi-square differences maybe expected due to young children’s characteristics. The theoretical moderation model fit both sample groups separately, and partial invariance (measures and structure) in multigroup comparison was observed, thus attesting for theoretical adequacy to predict poorer child adjustment in this early development phase when the child custody process in still ongoing in court.

## 4. Discussion

Within the scope of research on the impact of interparental conflict on child development, this study aimed to explore the combined predictive effects of authoritarian and permissive parenting styles and negative coparenting on psychosocial adjustment of young children of newly separated or divorced parents with an in-process child physical custody case in Family and Minors Court. In this study, we confirmed this theoretical moderation model for both younger and older groups of children with similarities between them. In line with previous studies, we found that post-divorced or separation parenting combined with negative, destructive coparenting can predict negative outcomes for children [13,14,15,16,17]. This relation was found for the two- or three-year-old group, indicating the risk of negative outcomes in early developmental stages.

Despite the equivalence of sole and joint physical custody groups, children spent, on average, more days per month with the mother than with the father. This information is consistent with 2019 national statistical data, which indicate the existence of more single parent maternal families than single father families [32], although they did not consider civil unions legally recognized. As with other western countries, household arrangements and overnights are not symmetric. The Portuguese family law changed in 2008 and had additional updates, and, since then, both parents have the legal right to share custody and should equally participate in the child’s upbringing, sharing parental responsibilities regardless of the parents’ previous relationship (no relationship, married, or cohabiting) or type of residence arrangement (joint or sole). Nowadays, it is possible to initiate a process of mutual agreement on parental responsibilities in a simplified way. However, it must always be submitted to the Family and Minors Court for approval or hearing, if necessary, except family circumstances in which it is not possible or when it is not in the best interests of the child. Notwithstanding this, household arrangements remain asymmetric as in other western countries [33,39]. Even with equivalent groups of sole and joint psychical custody, the distribution of the number of days with fewer days spent with the father follows that found in earlier studies Based on attachment to the mothers, children under three are traditionally in sole custody, spending fewer overnights with the father, thus overlooking attachment to fathers or father involvement [14,55]. All indicators are relevant for understanding young children’s best interest, wellbeing, and adjustment and should be considered in child physical custody decisions. According to the resilient risk framework, if the child is exposed to harsh parenting, interparental conflict, and triangulation from a young age, he is expected to be more vulnerable and develop negative outcomes and lower psychosocial adjustment, particularly in earlier stages of development [12,13,15,16,17,31].

In this study, we tested the moderation path model of parenting and coparenting negative variables (authoritarian, permissive, conflict, and triangulation) as predictors for the child psychosocial adjustment from the parents’ perspective. The theoretical independent moderation path models presented acceptable fit for data of the two groups, i.e., parents with 2- or 3-year-old children (group 1) and parents with children over three (group 2), similar to the theoretical common model. These results confirm our moderation hypothesis, whereby permissive and authoritarian parenting styles with conflict and triangulation predict poorer child adjustment [5]. Opposite to what was expected given previous studies, permissiveness seemed to act as a buffer and correlated in a significantly negative way with lower relational and emotional problems, hyperactivity, conduct problems, and lower triangulation for both groups. Similar results were found in previous studies in Southern European (e.g., Spain) and Southern American countries, implying the possible effect of contextual cultural aspects of the parenting styles concept [6]. Collectivist cultural values are pointed to as one possible explanation for cultural differences for indulgent permissiveness (warmth but low on control/demandingness) acceptation and outcomes [6]. Nonetheless, we found permissiveness together with authoritarian parenting style and negative coparenting to predict child maladjustment for both age groups. Our results are consonant with the spillover hypothesis of constant family systems interactions in family life, exhorting an effect on the children’s psychosocial adjustment. It may also reveal the inconsistency of parenting styles, which, as shown in previous studies, may create insecurity and vulnerability as well as psychological adjustment problems [6,8,35,57,58,66].

The multigroup comparison hypothesis was partially confirmed, attesting for the invariance of the prediction effect of parenting and coparenting in both measures and structure. The theoretical moderation path model fit both sample groups, showing the negative predictive effect of parents’ authoritarian, permissive, triangulation, and conflict on child adjustment for both younger and older children, only with little differences between them. This may be expected considering young children’s characteristics and needs. Permissive and authoritarian parenting styles, negative coparenting triangulation, and conflict have a negative effect for both younger and older children but may interact differently according to children’s age. Young children need more support, affection, and responsiveness and may feel parental conflict and triangulation differently. Although parenting demands may differ according to the children’ age and the developmental stage, harsh parenting and negative coparenting after separation or divorce with a child physical custody process undergoing in court affect the psychosocial adjustment of all children from infancy to adolescence. This is consonant with previous findings where coparenting acts as a mediator or moderator between parents’ communication in conflict and child maladjustment [66]. When the family emotional environment is embedded in destructive interparental conflict, restrictive and protective gatekeeping may occur, especially when younger children are involved [30,44,45].

Recently separated or divorced parents, friendly custody agreements included, may find it difficult to separate ex-marital issues from child rearing coparenting responsibilities, escalating and prolonging conflict over time [3]. Our study’s findings highlight the pathway moderation model of the mutual influence of authoritarian and permissive parenting styles and negative coparenting of conflict dimensions and triangulation on poorer child adjustment in early development. Although the sample size was not sufficient to separate between infancy, preschool-age, school-age children, and adolescents, the multigroup analysis partially proved the invariance of the model in two- or three-year-old children and children over three. Parenting and negative coparenting variables predicted lower levels of global child adjustment (relational problems, emotional symptoms, hyperactivity, and conduct problems) for both age groups. If experiencing destructive interparental conflict in infancy, children may display negative emotional reactions and integrate negative representations of interparental relationships with low emotional security, with proven longitudinal effects on preschool years such as conduct problems or aggressive behaviors [41,42,43,44]. Toddlers are sensitive to parental interaction, parent–child interactions transmitted through verbal and nonverbal communication. and child rearing practices that contribute to the family’s emotional climate and the child’s emotional security [11,24,25,46]. Despite their early development stage and inability to process conflict and negative parenting information, children are not alienated or protected from hazard. On the contrary, it can increase their vulnerability and risk of developing negative outcomes yet in different ways than older groups.

### 4.1. Strengths and Limitations

The key strength of this study was the focus on the transactional nature of interparental processes in a particularly sensitive and stressful couple’s separation or divorce phase regarding young children. Testing the parenting styles, the negative coparenting covariates, and the uniqueness of the data collection timeframe is an important insight to understand the process of adaptation of both parents and children to parents’ separation or divorce.

This study had several limitations, whereby results should be interpreted carefully. To ensure the feasibility of the use of questionnaires, we did not measure relevant control variables such as parents’ psychopathological indicators. The cross-sectional nature of the study precludes the generalization of the findings. Additionally, the sample size was not sufficient to test the path moderation model across different segmented age groups. Apart from the two- or three-year-old group, only the main age sample group included a wide age range and, consequently, possible confounding variables intrinsic to each developmental phase. Future studies should enlarge the sample size recruited in court, assuring equal age groups. In the future, ongoing child physical custody processes in family courts should continue to be addressed, if possible, including post-divorce/separation data in a longitudinal design. Upcoming studies should test the child adjustment model while controlling family bias by testing actor–partner effects model on child adjustment. For this article, we used parenting and negative coparenting types to clarify these possible age differences regarding the covariates’ predictive effect on child adjustment. In future studies, mother and father reports and more sensitive measures on parenting dimensions (responsiveness and demandingness) should be used. Additionally, other variables such as coping, emotional regulation strategies, and child observations or developmental indicators should shed light on the debate about what works in the child’s best interest and better adjustment, specifically in early childhood.

### 4.2. Conclusions and Implications

The main contribution of this study was to provide new data on parenting and coparenting predictive effects on young children’s psychosocial adjustment in a particularly stressed period of divorce/separation, a time propitious for conflict to occur, escalate, and become hard to manage, particularly in a court hearing. Summarizing the impact of parents’ separation or divorce and interparental conflict on children’s adjustment is complex and difficult to address in a single moment. Nonetheless, accounting for all limitations, the results presented here provide relevant and unique information regarding early child adjustment to parents’ parenting and coparenting when newly separated or divorced. The moderating model of the effect of less responsive or harsh parenting styles along with negative coparenting with conflict and triangulation predicts negative effects on child adjustment for both younger and older children of separated or divorced parents with an ongoing parental physical custody process in court. Although negative outcomes for both groups were confirmed, parenting and coparenting may interact differently according to child’s age (multigroup invariance was not confirmed for all models).

We hope to contribute to the discussion regarding young children’s adjustment to divorce, child custody, overnight plans, negative coparenting, and interparental conflict. Still, more research is needed. Regarding this study’s practical implications, judges, lawyers, social workers, psychologists, and caregivers, specifically parents involved in child custody decisions, should consider empirical findings regarding vulnerability and risk conditions for child adjustment in early developmental stages. Relevant decisions about young children’s lives should carefully consider parenting styles (observing responsiveness and demandingness/control) and coparenting styles with destructive conflict, triangulation, or constructive conflict. Moreover, other family variables (parents’ involvement, social support) and individual variables of parents (coping strategies, emotional regulation, stress) and children (development, attachment) should be considered. Understanding family emotional climate as well as individual and family processes of parenting and coparenting when dealing with a difficult divorce or separation phase is beneficial to understand children’s lower adjustment or negative outcomes. This study offers useful information to foster mediation processes and to help design multidisciplinary programs targeting separated or divorced parents that promote more adaptive and constructive approaches to managing conflict, hence helping mitigate negative psychological outcomes for the child and the parent–child relationship.

## Figures and Tables

**Table 1:** Descriptive statistics and correlation matrix of the variables according to children’s age (child 2/3 *n* = 93; child > 3 *n* = 114).

	Children > 3	1	2	3	4	5	6	7	8
Children 2/3									
*M*		9.74	20.43	13.57	25.4	2.59	4.22	2.65	3.34
*SD*		6.24	4.52	5.92	8.12	2.27	2.54	1.80	3.49
1. Triang.		1	−0.410 **	0.914 **	0.379 **	0.678 **	0.656 **	671 **	0.783 **
2. Perm.		−0.085 **	1	−0.372 **	−0.612 **	−0.320 **	−0.281 **	−0.234 **	−0.277 **
3. Conf.		0.905 **	0.006 **	1	0.343 **	0.710 **	0.647 **	607 **	0.753 **
4. Auth.		0.114 **	−0.640 **	0.083 **	1	0.541 **	0.629 **	0.549 **	0.477 **
5. RP		0.592 **	−0.264 **	0.566 **	0.338 **	1	0.766 **	0.763 **	0.764 **
6. Hyp.		0.579 **	−0.126 **	0.499 **	0.392 **	0.467 **	1	0.809 **	0.721 **
7. CP		0.589 **	−0.068 **	0.621 **	0.338 **	0.354 **	0.667 **	1	0.771 **
8. EP		0.664 **	−0.307 **	0.653 **	0.467 **	0.556 **	0.697 **	0.689 **	1
*M*		6.90	22.57	6.90	21.70	1.93	3.75	1.76	1.97
*SD*		4.08	3.54	4.82	6.47	1.67	1.60	1.31	2.13

Note. ** *p* < 0.01. Triang. = triangulation coparenting; Perm. = permissive parenting style; Conf. = conflict coparenting; Auth. = authoritarian parenting style; RP = relationship problems; Hyp. = hyperactivity; CP = conduct/behavioral problems; EP = emotional problems. Above the diagonal are the bivariate Pearson correlations for the group of parents with a child older than 3 years, and below are the bivariate correlations for parents with a 2- or 3-years old child.

**Table 2:** Results of multigroup analyses: standardized maximum likelihood estimate.

Models	CMIN/DF	CFI	PCFI	SRMR	RMSEA
Unconstrained	3.047 ***	0.960	0.446	0.0477	0.100
Measurement weights	2.778 ***	0.961	0.498	0.0486	0.093
Structural weights	2.772 ***	0.956	0.563	0.0560	0.093
Structural covariances	3.148 ***	0.930	0.714	0.1283	0.102
Structural residuals	3.206 ***	0.926	0.728	0.1278	0.104
Measurement residuals	3.309 ***	0.914	0.800		0.106
Saturated model		1.000	0.000		
Independence model	24.586 ***	0.000	0.000	0.1183	0.339

Note. *** significance at the level of *p* < 0.001; CFI = comparative fit index; PCFI = parsimony comparative fit index; TLI = normed fit index; RMSEA = root mean square error of approximation; SRMR = standardized root mean square residual.

**Table 3:** Full out study of significant difference between groups.

Model	DF	CMIN	*p*
Assuming model unconstrained to be correct:			
Measurement weights	3	1.346	0.718
Structural weights	7	12.263	0.092
Structural covariances	17	56.141	0.000
Structural residuals	18	61.860	0.000
Measurement residuals	23	82.930	0.000
Assuming model measurement weights to be correct:			
Structural weights	4	10.917	0.028
Structural covariances	14	54.795	0.000
Structural residuals	15	60.514	0.000
Measurement residuals	20	81.584	0.000
Structural weights	4	10.917	0.028
Assuming model structural weights to be correct:			
Structural covariances	10	43.878	0.000
Structural residuals	11	49.596	0.000
Measurement residuals	16	70.667	0.000
Assuming model structural covariances to be correct:			
Structural residuals	1	5.719	0.017
Measurement residuals	6	26.789	0.000
Assuming model structural residuals to be correct:			
Measurement residuals	5	21.070	0.001

## Data Availability

Unpublished data.
